# *SSU_RS09155*, a virulence-associated gene encoding a putative membrane of unknown function, that is essential for *Streptococcus suis* serotype 2 virulence

**DOI:** 10.1186/s13567-026-01761-7

**Published:** 2026-05-05

**Authors:** Servane Payen, Sonia Lacouture, Nahuel Fittipaldi, Mariela Segura, Marcelo Gottschalk

**Affiliations:** Research Group on Infectious Diseases in Production Animals (GREMIP) and Swine and Poultry Infectious Diseases Research Center (CRIPA), Department of Pathology and Microbiology, Faculty of Veterinary Medicine, University of Montreal, 3200 Sicotte St., Saint-Hyacinthe, QC J2S 2M2 Canada

**Keywords:** *Streptococcus suis*, virulence-associated gene, *SSU_RS09155*, murine and swine infection models, phagocytosis, bactericidal resistance, membrane protein

## Abstract

*Streptococcus suis* is an important swine pathogen and zoonotic agent, with the capsular polysaccharide recognized as its major virulence factor. Recent genomic studies have identified several virulence-associated genes (VAGs), including *SSU_RS09155*, *SSU_RS09525*, and *SSU_RS03100*, as potential markers of pathogenicity. The current study evaluated the direct contribution of these genes to *S. suis* virulence using isogenic deletion mutants of the serotype 2 reference strain P1/7. In a murine infection model, *ΔSSU_RS09525* and *ΔSSU_RS03100* mutants displayed virulence comparable to the parental strain, whereas the *ΔSSU_RS09155* mutant was significantly attenuated, showing reduced mortality and bacteremia. In vitro, the *ΔSSU_RS09155* mutant demonstrated increased susceptibility to killing in whole blood and enhanced phagocytosis by murine macrophages, while no significant defects in capsular polysaccharide (CPS) expression or growth in rich medium and mouse and porcine plasma were observed. Complementation restored the resistance to the whole-blood killing phenotype, confirming the specific role of *SSU_RS09155*. Adhesion to and invasion of porcine tracheal epithelial cells were unaffected. In pigs, the natural host, the *ΔSSU_RS09155* mutant was almost completely avirulent, with minimal to undetectable bacteremia. In silico analysis suggested that *SSU_RS09155* encodes a hydrophobic, membrane-associated protein potentially involved in resistance to innate immune mechanisms, although the real function remains unknown. Together, these findings identify *SSU_RS09155* as a critical virulence factor in *S. suis* serotype 2, whereas *SSU_RS09525* and *SSU_RS03100* appear dispensable under the experimental conditions tested.

**Supplementary Information:**

The online version contains supplementary material available at 10.1186/s13567-026-01761-7.

## Introduction

*Streptococcus suis* is naturally present in the porcine upper respiratory tract and is part of the normal flora [1]. However, it is one of the most important pathogens of pigs, frequently causing meningitis, sudden death, and arthritis, among other infections, in nursery animals [1]. In the Netherlands, it is responsible for one third of antimicrobial use in the post-weaning period in pig farms [2]. It is also an important zoonotic agent mostly in Southeast Asian countries [3]. The majority of porcine *S. suis* infections are caused by strains of a relatively reduced number of serotypes [4]. Although the distribution of serotypes from clinical cases differs depending on the geographic location, serotype 2 strains are responsible for most cases in both swine and humans worldwide [5]. Besides this serotype, recent years have seen the emergence of serotype 9 strains among swine diseases in several European countries [4].

Although knowledge on the pathogenesis of the infection has significantly increased in the last years (especially for serotype 2 strains), additional research is still required to identify key factors involved in virulence [5]. There is no generalized agreement defining a strain as virulent and many confounding factors may influence the level of virulence of a given strain [5, 6]. The capsular polysaccharide (CPS) is a key proven virulence factor [5] but encapsulation does not automatically result in virulence, since non-virulent strains may also be encapsulated [5]. The terms “virulence factors” and “virulence markers” have been used interchangeably in *S. suis* research. However, studies with isogenic mutants have shown that “traditional virulence factors”, including the muramidase-released protein (MRP), the extracellular protein factor (EF), and the suilysin (SLY), serve better as markers of virulence, i.e., useful for pathotypes differentiation in certain serotypes, but may not be key players in driving virulence themselves [7, 8]. Furthermore, at least two of these markers (EF and SLY) are mostly absent in most North American serotype 2 strains recovered from diseased animals [9].

The increasing accessibility of next-generation sequencing technologies, along with the generation of large-scale data, has spurred the development of numerous programs and software tools for bacterial typing. Indeed, pan-genome analysis provides an in silico approach for the discovery of genes involved in the pathogenesis in bacterial pathogens, called virulence-associated genes or VAGs. Several studies have proposed different *S. suis* VAGs based on the study of isolates recovered from either diseased or clinically healthy animals, with most of them classifying such isolates as pathogenic, opportunistic, or non-pathogenic [10–12]. As mentioned above, and even though an important diversity among *S. suis* strains has been observed, most studies on virulence factors and the pathogenesis of the infection are based on archetypal serotype 2 strains, either virulent sequence (ST) type 1 European strains or epidemic ST7 strain [5]. Interestingly, North American serotype 2 strains are phenotypically and genotypically different from their Eurasian counterparts [5]. In recent years, pangenome analysis was carried out with a collection of field strains from North America, mainly the USA [13]. In that study, three accessory genes, corresponding to *S. suis* reference strain P1/7 (ST1) markers *SSU_RS09525*, *SSU_RS09155*, and *SSU_RS03100* were identified as having a significant association with the pathogenic pathotype (96% of North American isolates were positive), suggesting a novel genotyping scheme for predicting the pathogenicity of *S. suis* [13].

We hypothesized that the VAGs *SSU_RS09525, SSU_RS09155*, and *SSU_RS03100* represent true *S. suis* serotype 2 virulence factors. To test this hypothesis, we constructed isogenic mutants lacking each gene using a virulent serotype 2 strain P1/7 as wild type, and characterized them using and tested in several relevant in vitro and in vivo infection models.

## Materials and methods

### Ethics statement

This study was carried out in accordance with the recommendations of the guidelines and policies of the Canadian Council on Animal Care and the principles set forth in the Guide for the Care and Use of Animals. The protocols and procedures were approved by the Animal Welfare Committee of the University of Montreal (permit number Rech-1570).

### Bacterial strains and growth conditions

The strains and plasmids used in this study are listed in Table 1. Although the VAGs tested in the current study were originally described to be present in North American strains, they are also present in ST1 European-type strains [11]. Hence, the classical virulent European reference P1/7 strain (wild type) was used throughout this study (including for construction of the isogenic deficient mutants) because its experimental virulence is well known [14]. *S. suis* strains were cultured in Todd–Hewitt broth (THB; Becton Dickinson, Mississauga, ON, Canada) as previously described [15]. *E. coli* strains were also grown on as previously described [15]. For in vitro cell culture assays, bacteria were prepared as previously described [16] and re-suspended in cell culture medium. When needed, antibiotics (Sigma-Aldrich, Oakville, ON, Canada) were added to the media at the following concentrations: for *S. suis*, spectinomycin (Spc) at 100 μg/mL and for *E. coli*, kanamycin (Km) and spectinomycin at 50 μg/mL and ampicillin (Ap) at 100 μg/mL.

**Table 1:** **List of strains and plasmids used in this study**

Strain or plasmid	Characteristics	References
*Streptococcus suis*		
P1/7	Virulent serotype 2 ST1 strain isolated from a case of pig meningitis in the UK	[17]
P1/7 Δ*SSU_RS09155*	Isogenic mutant derived from P1/7; in frame deletion of *SSU_RS09155* gene	This study
P1/7 Δ*SSU_RS09525*	Isogenic mutant derived from P1/7; in frame deletion of *SSU_RS09525* gene	This study
P1/7 Δ*SSU_RS03100*	Isogenic mutant derived from P1/7; in frame deletion of *SSU_RS03100* gene	This study
P1/7 Δ*cpsF*	Isogenic non-encapsulated mutant derived from P1/7; in frame deletion of *cpsF*	[18]
P1/7 comp Δ*SSU_RS09155*	Δ*SSU_RS09155* mutant complemented with pMX1-*9155* complementation vector	This study
*Escherichia coli*		
TOP10	*F-mrcA* Δ(*mrr-hsdRMS-mcrBC*) *φ80 lacZΔM15 ΔlacX74 recA1 araD139* Δ*(*araleu) *7697 galU galK rpsL (StrR) endA1 nupG*	Invitrogen
MC1061		
	Host for pMX1 derivatives	[19]
Plasmids		
pCR2.1	Apr, Kmr, pUC ori, lacZΔ*M15*	Invitrogen
pSET4s	Spcr, pUC ori, thermosensitive pG + host3 ori, lacZΔ*M15*	[20]
pMX1	Replication functions of *pSSU1, MCS pUC19 lacZ SpR*, *malX* promoter of *S. suis*, derivative of pSET2	[20, 21]
p4Δ*SSU_RS09155*	pSET-4 s carrying the construct for *SSU_RS09155* allelic replacement	This study
p4Δ *SSU_RS09525*	pSET-4 s carrying the construct for *SSU_RS09525* allelic replacement	This study
p4Δ *SSU_RS03100*	pSET-4 s carrying the construct for *SSU_RS03100* allelic replacement	This study
pMX1-*SSU_RS09155*	pMX1 carrying intact *SSU_RS09155* gene	This study

### DNA manipulations

Genomic DNA was extracted from the *S. suis* wild-type strain using In-staGene Matrix solution (BioRad Laboratories, Mississauga, ON, Canada). Mini preparations of recombinant plasmids were carried out using the QI-Aprep Spin Miniprep Kit (Qiagen, Valencia, CA, USA). Restriction enzymes and DNA-modifying enzymes (Fisher Scientific, Ottawa, ON, Canada) were used according to the manufacturer’s recommendations. Oligonucleotide primers (Table 2) were obtained from Integrated DNA Technologies (Coralville, IA, USA) and polymerase chain reaction (PCRs) carried out with the iProof proofreading DNA polymerase (BioRad Laboratories) or the Taq DNA polymerase (Qiagen). Amplification products were purified using the QIAquick PCR Purification Kit (Qiagen) and sequenced using an ABI 310 Automated DNA Sequencer and ABI PRISM Dye Terminator Cycle Sequencing Kit (Applied Biosystems, Carlsbad, CA, USA).

**Table 2:** **List of oligonucleotide primers used in this study**

Name	Sequence (5′ –3′)	Construct
*SSU_RS09155*-ID1	CACGATTGCTTGCTTGAAAG	p4Δ*SSU_RS09155*
*SSU_RS09155*-ID2	CGGTGATTCTGGGTAATAAGG	p4Δ*SSU_RS09155*
*SSU_RS09155*-ID3	AAGACCAAAACCGCCAGTC	p4Δ*SSU_RS09155*
*SSU_RS09155*-ID4	GGCAATCATATTGTCGGTGC	p4Δ*SSU_RS09155*
*SSU_RS09155*-ID5	AGTCTGCAAAGACATTGAAGC	p4Δ*SSU_RS09155*-ID5
*SSU_RS09155*-ID6	CAGGAAATGAGGTAGGAAGATGACGGGTGATATGGCATTTTC	p4Δ*SSU_RS09155*-ID5
*SSU_RS09155*-ID7	GAAAATGCCATATCACCCGTCATCTTCCTACCTCATTTCCTG	p4Δ*SSU_RS09155*-ID5
*SSU_RS09155*-ID8	TGTTATCCGAACCAGCCAG	p4Δ*SSU_RS09155*-ID5
*SSU_RS09525*-ID1	ACCTGTTTCCAAGCCAAAG	p4Δ*SSU_RS09525*
*SSU_RS09525*-ID2	AGAGAGGCGAAACCAGAAG	p4Δ*SSU_RS09525*
*SSU_RS09525*-ID3	AAAAACGGATGCGATCTCC	p4Δ*SSU_RS09525*
*SSU_RS09525*-ID4	GGAAGAATACTTGCGTGACC	p4Δ*SSU_RS09525*
*SSU_RS09525*-ID5	ATATTACGCGATGGAGCCC	p4Δ*SSU_RS09525*
*SSU_RS09525*-ID6	CGAGGTATTGAGATGGTTCATGAACAGTATGGTGCAGTGG	p4Δ*SSU_RS09525*
*SSU_RS09525*-ID7	CCACTGCACCATACTGTTCATGAACCATCTCAATACCTCG	p4Δ*SSU_RS09525*
*SSU_RS09525*-ID8	TTAGATGTGCTGGAAGCGG	p4Δ*SSU_RS09525*
*SSU_RS03100*-ID1	CTGTTACTACGTTTCCGATAGG	p4Δ*SSU_RS03100*
*SSU_RS03100*-ID2	GAAAACCATGACGAAGTTGTTC	p4Δ*SSU_RS03100*
*SSU_RS03100*-ID5	GCAGTTGTAAATGCAGAGCAG	p4Δ*SSU_RS03100*
*SSU_RS03100*-ID6	CTACTTAACACCATCCAACTCCAAAAGCCATAGCACTCAAACC	p4Δ*SSU_RS03100*
*SSU_RS03100*-ID7	GGTTTGAGTGCTATGGCTTTTGGAGTTGGATGGTGTTAAGTAG	p4Δ*SSU_RS03100*
*SSU_RS03100*-ID8	CTGAGTATTTCTCATTGCAGAGC	p4Δ*SSU_RS03100*
pMX1-*SSU_ RS09155*-F	CCGCCATGGTTTGTCCTACAGAGGAGCC	pMX1*SSU_RS09155*
pMX1- *RS09155*-R	CGCGAATTCACTCACAAGGAAGTCCACG	pMX1-*SSU_RS09155*

### Construction of virulence-associated gene (VAGs)-defective mutants

Precise in-frame deletion of *SSU_RS09525*, *SSU_RS09155*, and *SSU_RS03100* genes from strain P1/7 were constructed using splicing-by-overlap-extension PCRs as previously described [14]. Overlapping PCR product were cloned into pCR2.1 (Invitrogen, Burlington, ON, Canada), extracted with *EcoRI*, recloned into the thermosensitive *E. coli*–*S. suis* shuttle plasmid pSET4s, and digested with the same enzyme, giving rise to the knockout vector *p4ΔcpsG*. Electroporation of wild-type strain P1/7 procedures for isolation of the mutant were previously described [22]. Allelic replacement was confirmed by PCR and DNA sequencing analyses. Amplification products were purified with the QIAgen PCR Purification Kit (Qiagen) and sequenced as described above.

### Complementation of the *SSU_RS09155*-defective mutant

The *pMX1* vector was used for the generation of recombinant plasmids for complementation analysis (Table 1). This vector is a derivative of the *E. coli*–*S. suis* shuttle cloning vector pSET2 [15] and possesses the *S. suis malX* promoter for transgene expression in *S. suis*. The entire *SSU_RS09155* gene was amplified from genomic DNA of *S. suis* P1/7 strain and cloned into pMX1 via *EcoRI* and *NcoI* sites, generating complementation vector pMX1-9155. This plasmid was introduced into *E. coli* MC1061 for verification of the sequence and then into the deletion mutant *SSU_RS09155* derived from *S. suis* P1/7 to construct *SSU_RS09155*-complemented mutants.

### Bacterial surface hydrophobicity assay

Relative surface hydrophobicity of the *S. suis* has been used as an indication of the expression of the CPS at the bacterial surface. Hydrophobicity of the wild-type and all mutant strains was determined by measuring adsorption to n-hexadecane as previously described [23]. To confirm the presence of the CPS in the obtained mutants, a coagglutination test (which recognizes the CPS at the bacterial surface) was also performed as described [24] with an antiserum and a monoclonal antibody [25] raised against the capsular polysaccharide of *S. suis* serotype 2.

### Bacterial growth analysis

Bacterial growth experiments were performed in microtubes (500 μL culture volume). A bacterial overnight culture grown (1 × 10^4^ CFU/mL) in THB was used to inoculate THB, mouse, or porcine plasma. Growth was followed during 24 h of incubation at 37 °C. The total numbers of CFU/mL were evaluated at different incubation times by plating samples on Todd–Hewitt agar (THA).

### *S. suis* virulence mouse model of systemic infection

This study was carried out in accordance with the recommendations of the guidelines and policies of the Canadian Council on Animal Care and the principles set forth in the Guide for the Care and Use of Laboratory Animals, including euthanasia to minimize animal suffering by the use of humane end points, applied throughout this study when animals were seriously affected (mortality was not an endpoint measurement). A standardized C57BL/6 J mouse model of infection was used [26]. Thirty 6-week-old female C57BL/6 J (Jackson Research Laboratories, Bar Harbor, ME, USA) were used for these experiments: 15 mice per group infected with either P1/7 wild-type or *ΔSSU_RS09155, SSU_RS09525* and *SSU_RS03100* mutant strains. Early stationary phase bacteria were washed twice in phosphate-buffered saline, pH 7.4, and resuspended in THB. Bacterial cultures were diluted and plated on THB agar (THA) to accurately determine bacterial concentrations. Mice were inoculated with 1 × 10^7^ CFU of wild-type or mutant strains via the intraperitoneal route and health and behavior monitored at least thrice daily until 72 h post-infection and twice thereafter until the end of the experiment (12 days post-infection) for the development of clinical signs of sepsis, such as depression, swollen eyes, rough hair coat, prostration, and lethargy and meningitis. For bacteremia studies, blood samples were collected from the caudal vein of surviving mice 12 h and 24 h post-infection and plated as previously described [26].

### Whole blood bactericidal (killing) assay

Following the results obtained in vivo with the mouse model of infection (see “Results” section), the rest of the experiments done in this study were carried-out with the wild-type and *ΔSSU_RS09155* mutant strains only. Blood was collected from 6- to 10-week-old mice, and the test was performed as previously described, with a few modifications [22]. Briefly, blood containing leukocytes (9 × 10^6^ cells/mL on average) were transferred to a microtube containing around 1 × 10^7^ CFU/mL of the wild-type or *ΔSSU_RS09155* mutant *S. suis* strains [multiplicity of infection (MOI) = 1] and incubated for 2 h, mixing every 20 min. After incubation, cells were lysed, and appropriate dilutions plated on THA to determine viable bacterial counts. Resistance to bacterial killing by blood leukocytes was compared with incubation in plasma alone (obtained by centrifuging whole blood at 1800 × g for 10 min at 4 °C). The percentage of bacteria killed was determined using the following formula: 1 − (bacteria in blood/bacteria in plasma) × 100%.

### Phagocytosis assay

J774A.1 murine macrophage (ATCC TIB-67; Rockville, MD, USA) were maintained in Dulbecco’s modified Eagle’s medium (Gibco, Burlington, ON, Canada) supplemented with 10% fetal bovine serum (Gibco) and grown at 37 °C with 5% CO_2_. Confluent cell cultures were scraped, seeded at 1 × 10^5^ cells/mL, and incubated for 3 h at 37 °C with 5% CO_2_ to allow cell adhesion. Cells were infected by adding 1 × 10^7^ CFU/mL of bacterial suspension (either the wild-type or the *ΔSSU_RS09155* mutant strain) in complete culture medium (MOI = 100), incubated for 1 h at 37 °C with 5% CO_2_, and phagocytosis assays performed as previously described using the antibiotic protection assay [22]. As a control, wells with media but without cells were used to ensure that final counts after 1 h of incubation were statistically similar between both strains, demonstrating no differences in growth in this medium at the incubation time used (control data not shown).

### Confirmation of lack of virulence of the Δ*SSU_RS09155* mutant *S. suis* strain using a swine model of systemic infection

A total of 24 recently weaned 3-week-old Landrace/white mixed-breed piglets were acquired from a commercial farm in Quebec, which had no history of endemic clinical problems caused by *S. suis*, had no vaccination program against this pathogen, and was free of porcine reproductive and respiratory syndrome virus. All animals were already colonized by *S. suis* or *S. suis*-like microorganisms, as they are part of the normal microbiota of the upper respiratory tract. Upon arrival, piglets were weighed, individually tagged, assigned to two groups (P1/7 wild-type or Δ*SSU_RS09155* mutant strain; *n* = 12 per group) with equal average weight (approximately 5–6 kg), and placed in the Level II experimental animal facility at the Faculty of Veterinary Medicine, University of Montreal. Piglets were fed with commercial, pelleted non-medicated food, with an addition of dry veggie supplements. After 3 weeks of acclimation, animals were inoculated intramuscularly (IM) with 1 mL containing 1 × 10^8^ CFU of *S. suis* serotype 2 strain P1/7 or the Δ*SSU_RS09155* mutant strain. For bacteremia studies, blood samples were taken at 24 h, 48 h, 72 h, and 96 h post-infection and plated as previously described [27]. Pigs were monitored three times per day over a period of 8 days for the presence of clinical signs and mortality. A daily clinical score was calculated on the basis of a clinical observation sheet as previously described by us [27]. Pigs having a clinical score = 3 in locomotion and general behavior for two consecutive days or a CNS clinical score = 3 were humanely euthanized. Euthanized and dead animals were given a score of 10. Ketamine (30 mg/kg; Narketan®, Vetoquinol, Lavaltrie, QC, Canada) and xylazine (5 mg/kg; Rompun®, Bayer, Mississauga ON, Canada) were administered IM to achieve complete anesthesia followed by intracardiac administration of pentobarbital sodium (60 mg/kg; Dorminal, Rafter 8 product, Calgary AB, Canada).

### Bacterial adhesion and invasion assays to porcine tracheal epithelial cells

The neonatal porcine tracheal epithelial cell line (NPTr), frequently used in *S. suis* studies, was used. Cells were cultured until confluence as previously described, then infected with 1 × 10^6^ CFU/well (MOI = 10) of the wild-type P1/7 or the *ΔSSU_RS09155 S. suis* strains, and incubated for 2 or 4 h at 37 °C in 5% CO_2_. The adhesion assay, which quantifies total cell-associated bacteria (surface-adherent and intracellular bacteria), and invasion assay (using the antibiotic protection assay) were both performed as previously described [20].

### In silico analysis

Secondary structural characteristics of the protein was determined using the Self-Optimized Prediction Method with Alignment (SOPMA) method [28]. It uses the amino acid sequence for characterization of the secondary structures such as the alpha helix, beta sheets, beta turn, and random coils. The three-dimensional (3D) structure of the targeted protein was not available in Protein Data Bank (PDB). Therefore, web tools AlphaFold and Robetta were used to determine the 3D model of the protein [29, 30].

The analysis of the hydrophobicity of the protein sequence was performed using the Protscale server [31], using the Kyte and Doolittle scale to assign a hydrophobicity score to each amino acid in the sequence. This scale ranges from negative values (indicating a hydrophilic nature) to positive values (indicating a hydrophobic nature). The grand average of hydropathy (GRAVY) was calculated by averaging the hydrophobicity scores of all amino acids in the sequence. A positive GRAVY score (> 0) indicates a globally hydrophobic protein, while a negative score (< 0) indicates a more hydrophilic protein.

### Statistical analyses

Normality of data was verified using the Shapiro–Wilk test. Accordingly, parametric (unpaired *t*-test) or non-parametric tests (Mann–Whitney rank-sum test), where appropriate, were performed to evaluate statistical differences between groups. For in vivo animal experiments, survival rates were evaluated with chi-squared analysis using the Kaplan–Meier method, and the significance of the difference was tested using the log-rank test. The clinical scores were transferred by ranking, and the significance of the difference between groups was determined by the Student’s test (or *t*-test) using GraphPad Prism 8 software (GraphPad Software, San Diego, CA, USA) to evaluate the significant difference between the mean values with a confidence interval set at 95%. Data were considered significantly different if *p* < 0.05.

## Results

### *SSU_RS09155*, *SSU_RS09525* and *SSU_RS03100* deletion mutants showed similar general characteristics to the P1/7 wild-type strain

**Figure 1:**
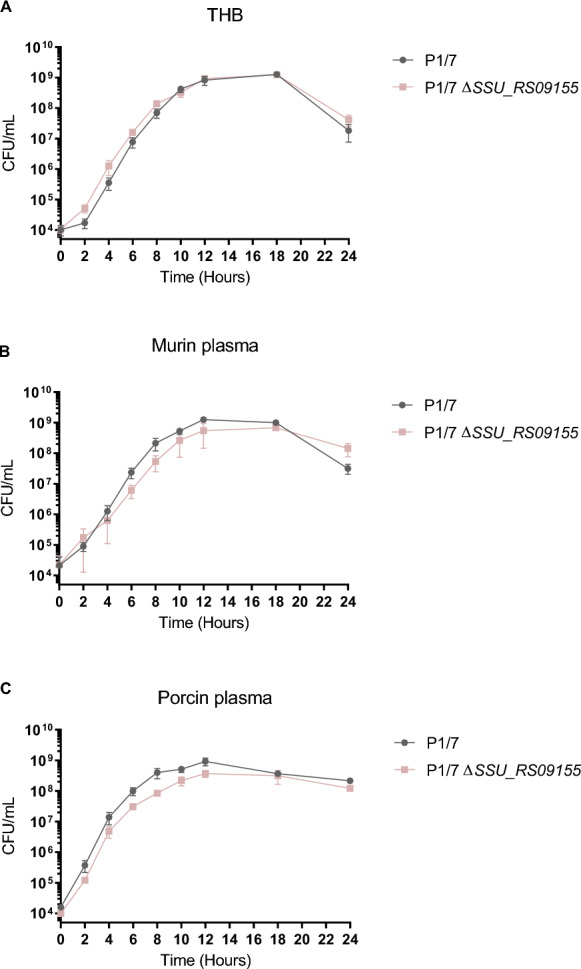
***ΔSSU_RS09155*** **mutant shows no growth defect in rich or plasma media**. *ΔSSU_RS09155* mutant has no statistically significant growth defects in rich and poor media. Growth of the wild-type P1/7 strain (black) and *ΔSSU_RS09155* (pink) in THB (**A**), murine plasma (**B**), and porcine plasma (**C**). *n* = 3 independent experiments.

The P1/7 *ΔSSU_RS09525*, *ΔSSU_RS09155*, and *ΔSSU_RS03100* mutants presented general phenotypic characteristics similar to the P1/7 wild-type strain. All mutants remained well encapsulated as shown by the presence of hydrophobicity values between 1.89% and 2.93%, similar to the wild-type strain (2.33%) and different from the non-encapsulated mutant used as a positive control (96.4%). In addition, mutants were similarly serotypable (serotype 2) by the coagglutination test using polyclonal and monoclonal antibodies. All mutant strains cultured in rich THB medium showed a normal growth similar to the P1/7 wild-type strain (Figure 1A for *ΔSSU_RS09155*; Additional file 1 for the other mutants). To mimic in vivo conditions, we also performed growth curves for the wild-type P1/7 and *ΔSSU_RS09155* mutant in murine and porcine plasma (Figure 1B, C), the natural environment for systemic invasion of *S. suis*. As previously described, the wild-type strain showed good growth in both murine and porcine plasma [22]. The growth of the *ΔSSU_RS09155* mutant, though slightly delayed between 6 and 12 h, was statistically similar to that of the wild-type strain during the entire incubation times.

### Presence of *SSU_RS09155*, but not *ΔSSU_RS09525* and *ΔSSU_RS03100*, is required for full virulence of *S. suis* in a mouse model of infection

**Figure 2:**
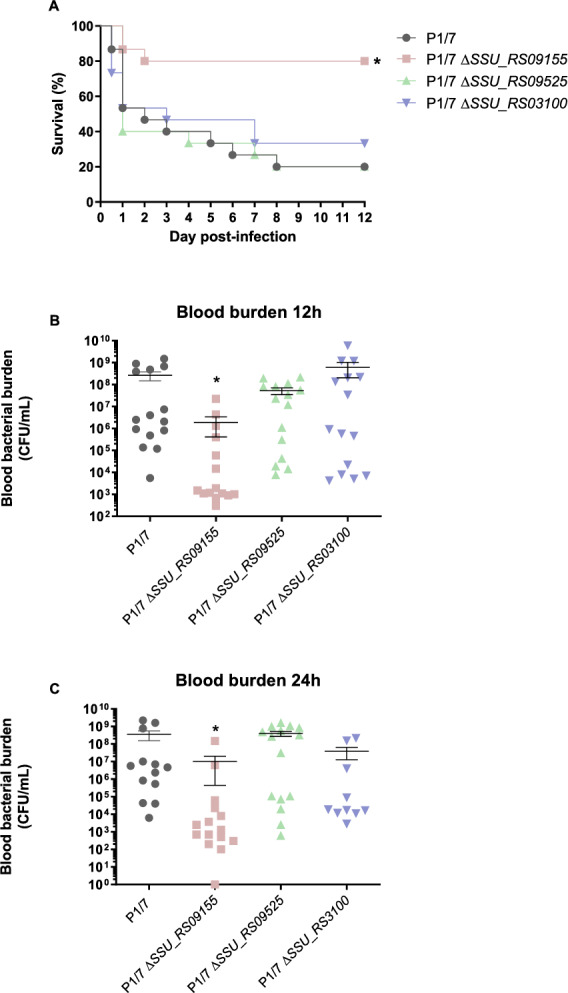
***SSU_RS09155***, **but not** ***SSU_RS09525*** or ***SSU_RS03100***, **is required for** ***S. suis*** **virulence in a mouse model of infection**. Presence of *VAG SSU_RS09155*, but not *SSU_RS9525* and *SSU_RS3100*, is required for *S. suis* systemic virulence and blood persistence following intraperitoneal inoculation of mice. Survival (**A**) and blood bacterial burden at 12 and 24 h post-infection (**B**, **C**) of C57BL/6 mice following intraperitoneal inoculation of the *S. suis* virulent wild-type P1/7 strain (black), *ΔSSU_RS09155* (pink), *ΔSSU_RS09525* (green), and *ΔSSU_RS03100* (bleu) mutant strains. Data represent survival curves (**A**) (*n* = 15) or geometric mean (**B**, **C**). ^*^p < 0.05 indicates a significant difference between survival or blood bacterial burden of mice infected either with the wild-type or the *ΔSSU_RS09155* mutant strain.

To investigate the role of the VAGs *SSU_RS09155*, *SSU_RS09525* and *SSU_RS03100* in the virulence of *S. suis* and the development of clinical disease, a well-characterized C56BL/6 murine infection model was used. Mice infected with the wild-type strain rapidly developed clinical signs of systemic disease characteristic of septic shock, with 46% of mice succumbing to the infection within 48 h (Figure 2A), similar to what has previously reported [20, 28, 30]. Similar results were observed with the *ΔSSU_RS09525* and *ΔSSU_RS03100* mutants, indicating that either of these genes does not seem to play an important role in virulence. However, only 20% of mice infected with the *ΔSSU_RS09155* mutant succumbed to the disease. All other mice developed transient signs of infection, such as rough fur following bacterial inoculation, but quickly recovered and exhibited normal behavior. These results suggest that the protein encoded by *SSU_RS09155* is important for the virulence of *S. suis* serotype 2 and may be not only associated with virulence but also involved in the pathogenesis of the infection.

Furthermore, bacterial load in the blood was also assessed at early infection time points of 12 and 24 h. Mice infected with the wild-type strain exhibited high bacterial loads in the blood, with an average higher than 1 × 10^8^ CFU/mL (Figure 2B, C). Similarly, *ΔSSU_RS09525* and *ΔSSU_RS03100* mutants displayed high bacterial loads similar to the wild-type strain at both time points post-infection. In contrast, mice infected with the *ΔSSU_RS09155* mutant showed a significant decrease in blood bacterial load at both 12- and 24-h post-infection. These data suggest that this mutant has a reduced capacity to survive in the bloodstream of infected mice, probably explaining the better survival of infected animals.

### Presence of *SSU_RS09155* is involved in resistance to bactericidal effect of blood and phagocytosis by macrophages

**Figure 3:**
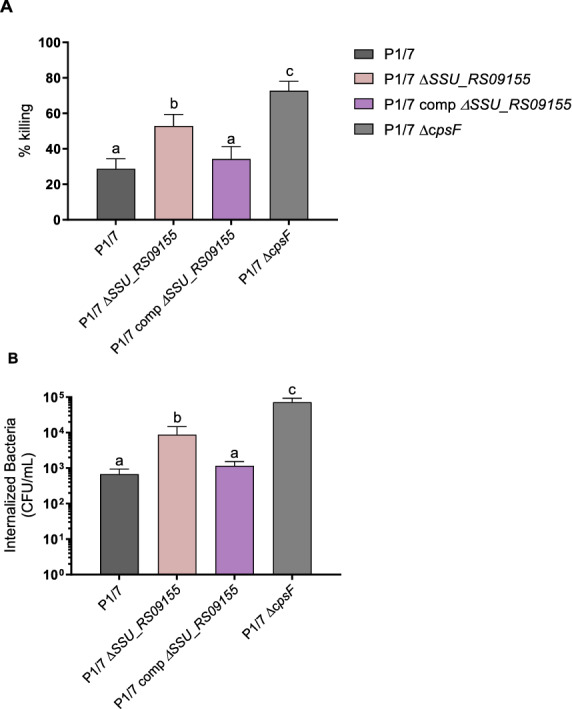
***SSU_RS09155*** **promotes resistance to whole blood killing and macrophage phagocytosis**. Presence of *SSU_RS09155* is required for *S. suis* whole blood bactericidal resistance and for anti-phagocytic properties. Capacity to resist the bactericidal effect of murine whole blood (**A**) and internalization by J774A.1 murine macrophages (**B**) of wild-type P1/7 (black), *ΔSSU_RS09155* (pink), P1/7 comp*ΔSSU_RS09155* (violet), and *ΔcpsF* (gray) mutant strains. Data represent the mean ± SEM (*n* = 3). ^*^Groups not sharing letters (a, b, or c) are significantly different from each other (*p* < 0.05).

Confirming results obtained during bacteremia after in vivo mouse infections, results showed that the *ΔSSU_RS09155* mutant was more susceptible to the in vitro bacterial killing effect of blood when compared with the wild-type strain (*p* = 0.03) (Figure 3A). A lower susceptibility was restored when the complemented P1/7 comp*ΔSSU_RS09155* mutant was used (Figure 3A), confirming the specific role of *SSU_RS09155*. As previously described [15, 22, 26], the non-encapsulated *ΔcpsF* mutant, used as a positive control, was also highly susceptible to the bacterial killing (*p* = 0.0002) (Figure 3A). Interestingly, although both *ΔSSU_RS09155* and *ΔcpsF* mutants were more susceptible to bacterial killing than the wild-type P1/7 strain, there was also a significant difference between them: the *ΔcpsF* non-encapsulated mutant was more susceptible than the *ΔSSU_RS09155* mutant (*p* = 0.02), confirming the predominant role of the CPS on the resistance to whole-cell blood killing.

Similar results were obtained with the phagocytosis studies with macrophages (Figure 3B). Both *ΔSSU_RS09155* and *ΔcpsF* mutants presented a higher phagocytosis rates than the wild-type P1/7 (*p* = 0.03 and 0.0002), with the *ΔcpsF* mutant being also higher phagocytosed when compared with the *ΔSSU_RS09155* mutant (*p* = 0.01). Similar to the killing test, the activity was restored when using the complemented P1/7 comp*ΔSSU_RS09155* mutant (Figure 3B).

### Lack of *SSU_RS09155* does not impair adhesion to and invasion of respiratory swine epithelial cells

**Figure 4:**
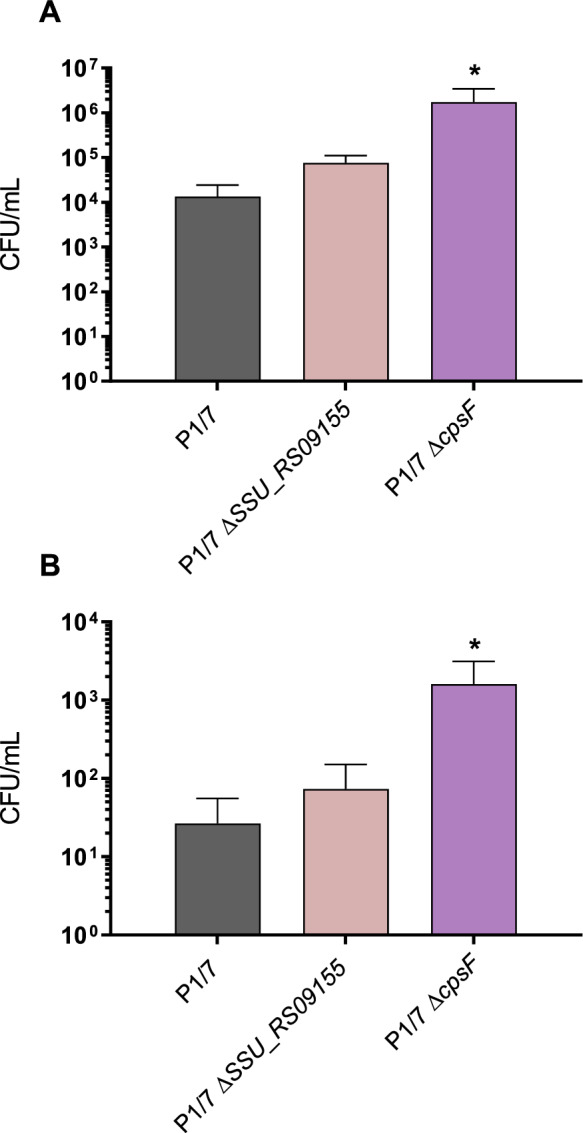
***ΔSSU_RS09155*** **deletion does not affect adhesion or invasion of epithelial cells**. The *ΔSSU_RS09155* mutant is not impaired in its capacity of adhesion to and invasion of epithelial cells. Adhesion (**A**) and invasion (**B**) of the *S. suis* serotype 2 wild-type virulent strain P1/7 (gray) and *ΔSSU_RS09155* (pink) to porcine tracheal epithelial cells after 2 h of incubation. ^*^p < 0.05 indicates a significant difference between the *ΔcpsF* mutant and wild-type strain. Each bar represents the mean bacterial concentration (CFU/mL) + SEM (*n* = 3 independent experiments).

The role of the proposed VAGs in the adhesion/invasion to NPTr cells was evaluated using the generated mutant strains. As expected, the non-encapsulated mutant strain used as a positive control significantly adhered to and invaded cells more efficiently than the wild-type strain (*p* = 0.02) (Figure 4A, B). However, the *ΔSSU_RS09155* mutant similarly adhered to (Figure 4A) and (weakly) invaded (Figure 4B) NPTr cells.

### Confirmation of lack of virulence of the *ΔSSU_RS09155* mutant *S. suis* strain using a swine model of systemic infection

**Figure 5:**
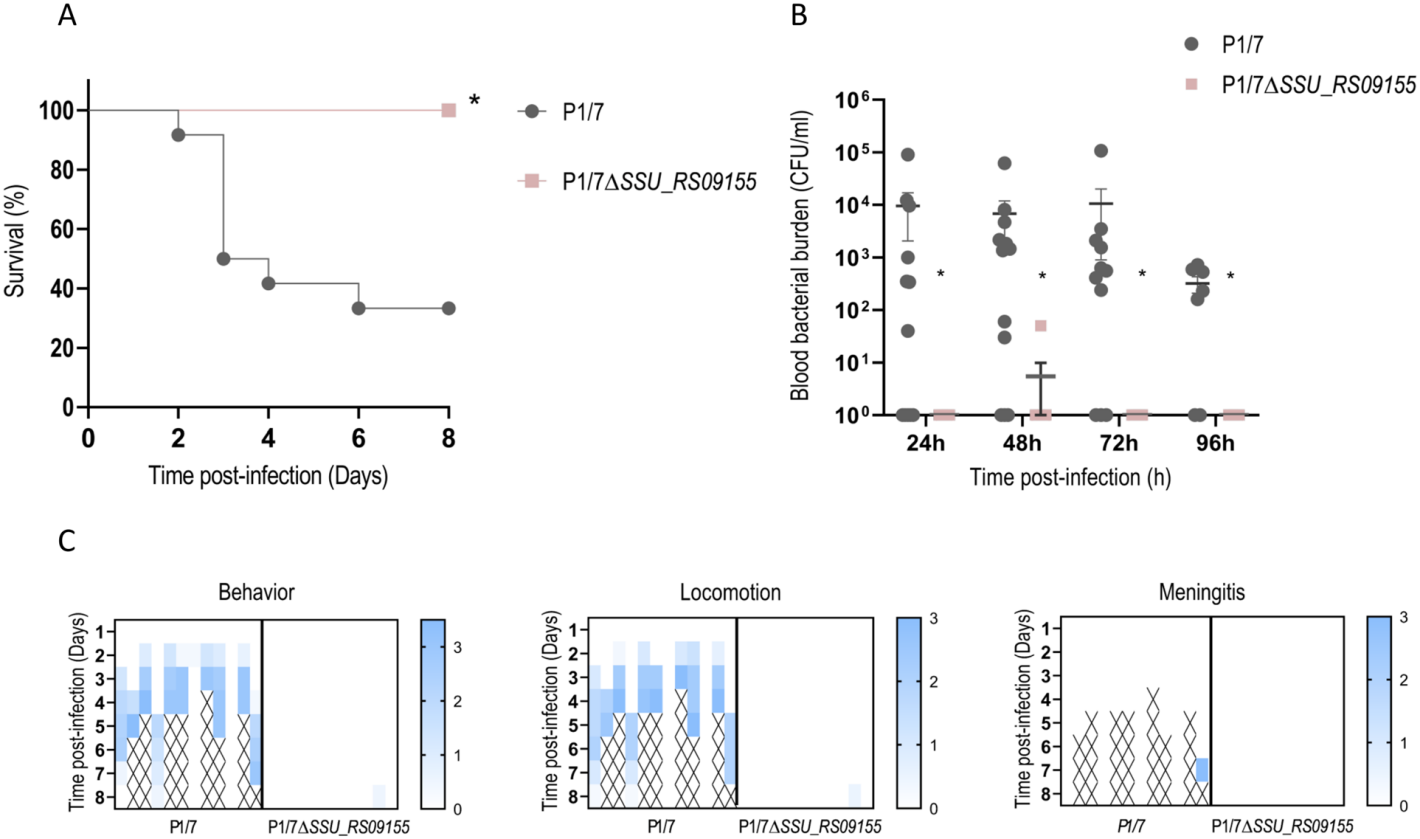
***SSU_RS09155*** **contributes to virulence and bacteremia during** ***S. suis*** **infection in piglets**. **A** Survival rates and **B** blood bacterial burden (**B**), following intramuscular infection of piglets with 1 mL containing 1 × 10^8^ CFU of a log-phase culture *S. suis* P1/7 (black) or the *ΔSSU_RS09155* mutant strain (pink). **C** Mean clinical scores. Pigs were monitored three times per day over a period of 8 days for the presence of clinical signs. A daily clinical score was calculated on the basis of general behavior, locomotion (musculoskeletal alterations), and central nervous system dysfunction, as described in the “Materials and Methods” section. The intensity of the blue color reflects the severity of clinical scores, with darker shades indicating more severe signs. A cross symbol indicates death or euthanasia.

One piglet in the group infected with the *ΔSSU_RS09155* became blue (hypoxia) and suddenly died during the infection procedure, and it was excluded from the study. The wild-type strain P1/7 caused 66.6% of mortality (8 pigs out of 12), similar to what has already been reported [27]. However, none of the animals from the group infected with the *ΔSSU_RS09155* mutant had to be euthanized (*p* < 0001) (Figure 5A). Wild-type P1/7 strain was recovered from blood samples of most infected animals during the first 96 h, whereas the mutant *ΔSSU_RS09155* was in general unable to invade and/or survive in the bloodstream (Figure 5B). Challenged animals showed signs of depression, incoordination, and shifting lameness. In more severe cases, there were signs of arthritis (locomotion problems) and meningitis, the latest characterized by convulsion, head inclination, ataxia, opisthotonos, paddling, and nystagmus (Figure 5C).

### In silico analysis suggests that the protein encoded by SSU_RS09155 is potentially located at the membrane level

The protein encoded by *SSU_RS09155* is annotated as a hypothetical protein in the *S. suis* genome, and as such, its role remains unknown. However, on UniProt, it is annotated as a membrane protein (Accession number: A0A0H3MXK8). Comparative genomic analyses have shown that SSU_RS09155 is widely distributed among *S. suis* strains [13]. To assess its broader distribution within the genus, BLASTP searches were performed using the SSU_RS09155 sequence as query, which identified homologous proteins in several other *Streptococcus* species (data not shown).

**Figure 6:**
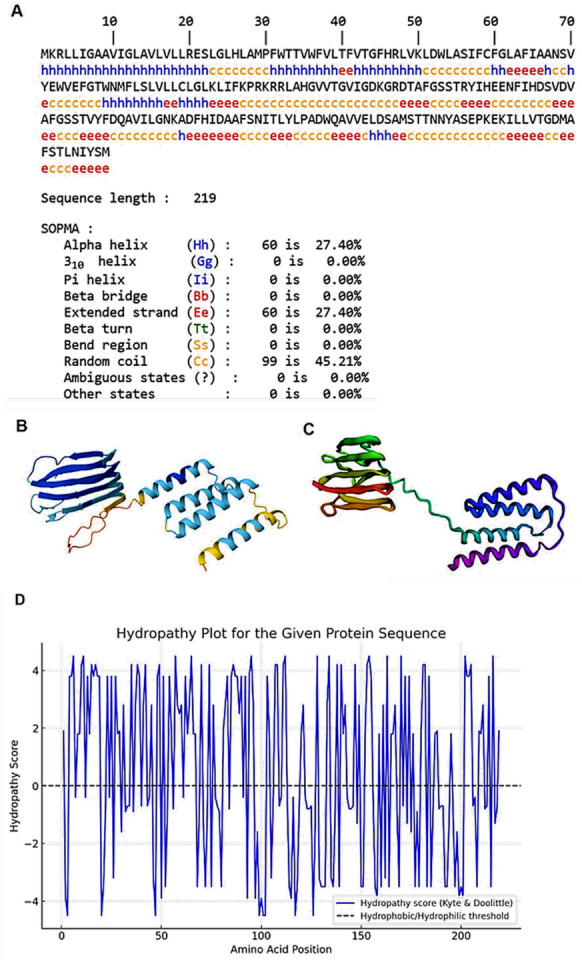
**Structural and physicochemical characterization of the SSU_RS09155 protein**. **A** Secondary structure prediction based on the SOPMA algorithm. The protein comprises 219 amino acids, with the following predicted elements: α-helices (blue, 27.40%), extended strands (red, 27.40%), and random coils (orange, 45.21%). The sequence and predicted secondary structures are shown in alignment. **B** Predicted 3D structure of the targeted protein generated by AlphaFold. **C** Alternative structural prediction obtained using Robetta. **D** Hydropathy plot of the protein sequence generated using the Kyte and Doolittle scale. Positive scores indicate hydrophobic regions, while negative scores indicate hydrophilic regions.

The sequence of proteins consists of 219 amino acids, with 24.4 KDa molecular weight. The protein’s secondary structure was determined using SOPMA. According to its analysis, the secondary structural elements consist of alpha helices, extended strands, and random coils. The results from SOPMA (Figure 6A) showed 60 amino acids in alpha helix (27.40%), 60 amino acids in extended strands (27.40%), and 99 amino acids in random coils (45.21%). No 3_10_ helix, pi helix, beta bridge, beta turn, or bend region was detected.

Following this analysis, the 3D structure of the proteins was modeled by AlphaFold (Figure 6B) and Robetta (Figure 6C). The SOMAP analysis and the 3D structure of 9155 suggest that it could be a protein with hydrophobic domains and, consequently, a membrane protein. Moreover, the hydropathy plot of the analyzed protein sequence, based on the Kyte and Doolittle scale, reveals multiple regions with positive hydropathy scores (Figure 6D). These peaks, exceeding a value of 0, indicate hydrophobic segments, which are characteristic of transmembrane domains or membrane-associated regions. Conversely, regions with negative hydropathy scores suggest hydrophilic domains, likely exposed to the aqueous environment. The overall GRAVY score of 0.52 supports the hypothesis that this protein is predominantly hydrophobic, further suggesting its potential localization in the membrane.

These findings align with the possibility that this protein may function as a membrane protein, possibly spanning the lipid bilayer or associated with it. Further validation using transmembrane domain prediction tools or experimental assays would be necessary to confirm its precise role and localization.

## Discussion

The identification and functional validation of virulence factors in *S. suis* remains essential for understanding the pathogenesis of the infection and for developing improved diagnostics and preventive tools. The diagnostic of *S. suis* as a primary pathogen is not an easy task, especially when clear high-virulence strains (such as those belonging to clonal complex 1 or CC1) are absent in the herd, and in addition, clear predisposal factors, such as the porcine reproductive and respiratory syndrome virus, are present [6]. This is usually the case in Canada and USA, where multiple serotypes (most of them not CC1) are isolated from diseased pigs, sometimes within the same farm [1]. Therefore, the identification of virulence markers (virulence-associated genes [VAGs]) able to differentiate virulent from commensal/opportunistic strains becomes highly important.

In recent years, there has been some confusion about VAGs. Definition of a VAG relies usually [32] on the presence of a gene in a virulent strain and its absence in a non-virulent one. However, the definition of a “virulent strain” is not easy for *S. suis* [5]. In fact, virulent strains may be isolated from clinically healthy animals and, in some cases, strains recovered from diseased animals may behave as non-virulent after experimental infection, even with highly susceptible cesarean-derived, colostrum-deprived pigs [33]. It is well known that not only the virulence potential of the strain involved but also the environmental and management factors as well as the presence of co-infections, may have a significant influence on the appearance of clinical signs due to *S. suis* [6]. Despite these difficulties, several VAGs schemes have been described for this pathogen [11, 32–36]. At least one of the proposed VAG classifications [35] was shown not to be useful when applied to Swiss and Canadian field isolates ([37] and Gottschalk, unpublished data, respectively).

It has been previously shown for *S. suis* that a VAG is not necessarily a critical virulence factor. For example, as previously mentioned, the “traditional” virulence markers MRP, EF, and SLY (encoded by *mrp*, *epf*, and *sly* genes) usually associated to highly virulent strains are not critical virulence factors [7, 8]. Interestingly, most VAGs have been described for Eurasian strains. One study described three VAGs—*SSU_RS09155*, *SSU_RS09525*, and *SSU_RS03100*—associated with pathogenic strains, particularly from North America [13]. In the current study, we evaluated the virulence potential of isogenic mutants defective in each of those VAGs. We used the well-characterized European CC1 virulent reference strain P1/7 as wild type since these VAGs were also present in such strain.

Results showed that only *SSU_RS09155* encoded for an important virulence factor, while *SSU_RS09525* and *SSU_RS03100*, although associated with virulent strains in the original study, do not appear to play a direct role in virulence under our experimental conditions. Deletion of *SSU_RS09155* resulted in a clear attenuation of virulence in a murine infection model. More importantly, this mutant was shown to be avirulent in a well characterized swine model of infection.

Surface capsule expression, a critical virulence factor for *S. suis* serotype 2, was similar for the wild-type strain and the *SSU_RS09155* mutant strain. In general, bacterial growth was also similar to the wild type in rich (THB) medium and in murine/swine plasma. We observed a slight (nonsignificant) delay during the exponential phase of growth (first 12 h), but with identical bacterial counts at the stationary phase. Similar observations have recently been made by Juanpere-Borras et al. with a similar mutant; however, these authors tested the mutant in serum (not plasma), and the slight reduced growth was also observed at the stationary phase [38]. Results obtained in the current study indicate that the observed phenotype was most probably not due to general fitness defects but rather to a specific impairment in host interaction mechanisms. The mouse model of infection has been widely used to evaluate the virulence of *S. suis* mutant strains [39] [14, 38], although many studies have not confirmed results in a swine model of infection. In a few instances where both species have been used, the effect of a given mutation on virulence showed different results in mice and pigs [8, 40]. In the present study, results obtained were similar in both species.

In vitro assays provided additional insights into the role of *SSU_RS09155* in virulence. The deletion mutant displayed increased susceptibility to whole-blood killing and enhanced phagocytosis by murine macrophages, suggesting that the locus contributes to resistance against innate immune defenses. These phenotypes were significantly restored in the complemented mutant, confirming the specific contribution of the deleted gene. This may explain why pigs infected with the *SSU_RS09155* strain cleared the infection rapidly.

The main antiphagocytic virulence factor in *S. suis* is the CPS [41], which is also the most important determinant of resistance to the bactericidal effect of blood [42]. Nevertheless, several proteins have been reported to contribute to one or both of these properties [39, 43–51], although the underlying mechanisms have often not been clearly elucidated. In some cases, deletion of a gene encoding such a protein indirectly leads to downregulation of CPS expression, thereby reducing resistance to immune defenses [52–54]. Notably, at least one described protein has been shown to directly affect resistance to blood killing and phagocytosis without altering CPS production [54]. In the current study, the increased susceptibility of the *ΔSSU_RS09155* mutant to host defenses, while significant and not related to the absence/reduction of the CPS expression, was still less pronounced than that of a non-encapsulated control mutant. This finding reinforces the central, yet not exclusive, role of the CPS in immune evasion. However, *SSU_RS09155* does not seem to be involved in adhesion/invasion of epithelial cells, processes that represent the initial steps in the pathogenesis of the infection.

The exact mechanistic basis underlying the function of the *SSU_RS09155*-encoded protein remains unknown. In silico analyses suggest that it is a small, hydrophobic protein that may associate with the membrane, but these predictions do not provide functional insight. At present, other than the anti-phagocytic role hereby described, there is no evidence to determine exactly how this protein contributes to virulence, and experimental studies will be required to define its role.

Different from what was found with the *SSU_RS09155* mutant, deletion of *SSU_RS09525* and *SSU_RS03100* had no observable impact on virulence in the mouse model of infection. These results suggest that these genes, although statistically associated with pathogenic strains in genotyping studies, are not essential for virulence under the experimental conditions tested. It is possible that their association with pathogenicity reflects linkage to other functional loci or that their contribution is conditional, depending on strain background, host species, or route of infection.

## Conclusions

This study underscores the importance of experimentally validating genomic associations to distinguish true virulence factors from lineage- or context-dependent markers. Deletion of VAG *SSU_RS09155* results in clear attenuation of *S. suis* serotype 2 in both mouse and swine models, establishing this locus as a bona fide virulence factor under our experimental conditions. In contrast, deletion of *SSU_RS09525* or *SSU_RS03100*, despite their previously reported statistical association with pathogenic strains, had no detectable impact on virulence, indicating that these loci serve as virulence markers but are not factors. The increased susceptibility of the *SSU_RS09155* mutant to blood killing and phagocytosis indicates that this locus contributes, directly or indirectly, to innate immune resistance, but the underlying mechanism remains unknown. Extending this approach to other VAGs identified in large-scale genomic surveys could refine our understanding of the virulence repertoire of *S. suis* and help distinguish core virulence factors from lineage-specific markers.

## Supplementary Information

**Additional file 1.** ***ΔSSU_RS09525*** **and** ***ΔSSU_RS3100*** **mutants showed no growth defect in THB medium.** *ΔSSU_RS09525* and *ΔSSU_RS3100* mutants have no statistically significant growth defects in THB medium. Growth of the wild-type P1/7 strain (black), ΔSSU_RS09525 (green) and ΔSSU_RS3100 (blue) were statistically similar (*p* > 0.05). *n* = 3 independent experiments.

## Data Availability

No datasets were generated or analyzed during the current study.

## Abbreviations

AP: ampicillin
CPS: capsular polysaccharide
CFU: colony forming unit
EF: extracellular protein factor
Km: kanamycin
KDa: kilodalton
MOI: multiplicity of infection
MRP: muramidase-released protein
NPTr: neonatal porcine tracheal epithelial cell line
*S. suis*: *Streptococcus suis*
SOPMA: self-optimized prediction method with alignment
Spc: spectinomycin
ST: sequence typing
SLY: suilysin
THA: Todd–Hewitt agar
THB: Todd–Hewitt broth
VAG: virulence-associated gene
WT: wild type
