# Supplementary material for: SSU_RS09155, a virulence-associated gene encoding a putative membrane of unknown function, that is essential for Streptococcus suis serotype 2 virulence
Source: Vet Res. 2026 May 5;57:93. doi: 10.1186/s13567-026-01761-7 (PMC13214195; doi:10.1186/s13567-026-01761-7)
Supplement: Supplementary file 1 [file 13567_2026_1761_MOESM1_ESM.docx]

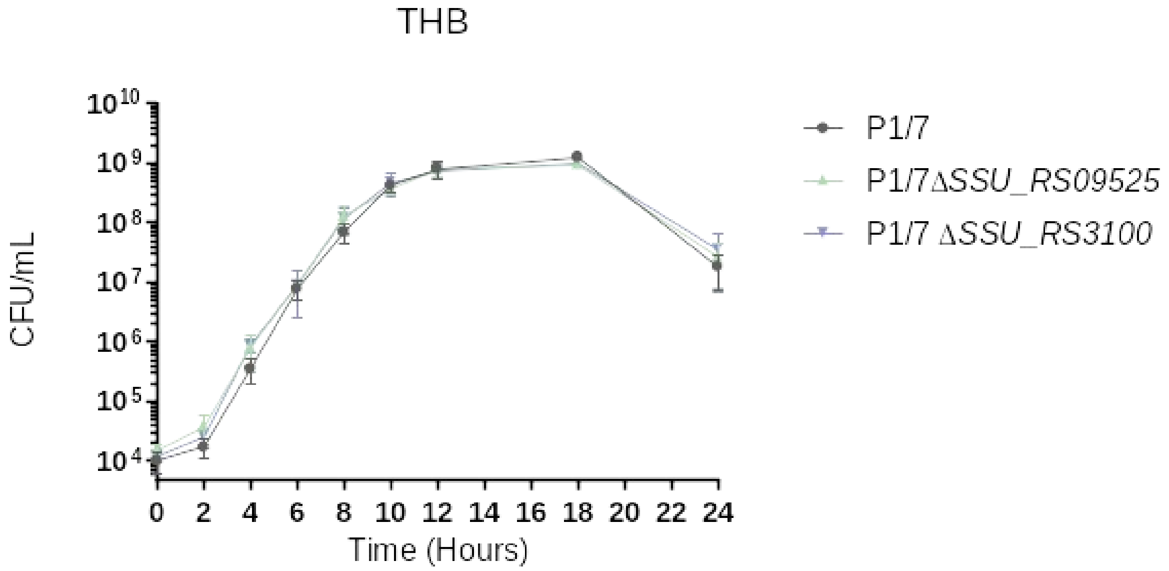
THB
CFU/mL
10^10
10^9
10^8
10^7
10^6
10^5
10^4
0 2 4 6 8 10 12 14 16 18 20 22 24
Time (Hours)
P1/7
P1/7ΔSSU_RS09525
P1/7 ΔSSU_RS3100
